# Interhemispheric Transfer Time Asymmetry of Visual Information Depends on Eye Dominance: An Electrophysiological Study

**DOI:** 10.3389/fnins.2018.00072

**Published:** 2018-02-16

**Authors:** Romain Chaumillon, Jean Blouin, Alain Guillaume

**Affiliations:** Aix Marseille Univ, CNRS, LNC, Laboratoire de Neurosciences Cognitives, Marseille, France

**Keywords:** eyedness, interhemispheric communication, corpus callosum, visually-evoked potentials, current source density analysis

## Abstract

The interhemispheric transfer of information is a fundamental process in the human brain. When a visual stimulus appears eccentrically in one visual-hemifield, it will first activate the contralateral hemisphere but also the ipsilateral one with a slight delay due to the interhemispheric transfer. This interhemispheric transfer of visual information is believed to be faster from the right to the left hemisphere in right-handers. Such an asymmetry is considered as a relevant fact in the context of the lateralization of the human brain. We show here using current source density (CSD) analyses of visually evoked potential (VEP) that, in right-handers and, to a lesser extent in left-handers, this asymmetry is in fact dependent on the sighting eye dominance, the tendency we have to prefer one eye for monocular tasks. Indeed, in right-handers, a faster interhemispheric transfer of visual information from the right to left hemisphere was observed only in participants with a right dominant eye (DE). Right-handers with a left DE showed the opposite pattern, with a faster transfer from the left to the right hemisphere. In left-handers, albeit a smaller number of participants has been tested and hence confirmation is required, only those with a right DE showed an asymmetrical interhemispheric transfer with a faster transfer from the right to the left hemisphere. As a whole these results demonstrate that eye dominance is a fundamental determinant of asymmetries in interhemispheric transfer of visual information and suggest that it is an important factor of brain lateralization.

## Introduction

The communication between the two hemispheres through the corpus callosum is a fundamental process in the human brain (see Gazzaniga, 2000; for a review). One crucial parameter of the transfer of information from one hemisphere to the other is its speed, referred to as the interhemispheric transfer time (IHTT). The prevailing theory of interhemispheric transfer of visual information, arising from both behavioral (Marzi et al., 1991; Braun, 1992) and electrophysiological (Saron and Davidson, 1989; Moes et al., 2007) investigations, posits that there is an asymmetry in IHTT with a faster interhemispheric transfer from the right to the left hemisphere than in the reverse direction. Despite not being clearly understood, this asymmetry has been seen as an important lateralization in the human brain (Marzi, 2010).

Surprisingly, although the lateralization of the visual system known as eye dominance has long been recognized (e.g., Wardrop, 1808), its potential role in visual IHTT asymmetry has remained unsuspected. The sighting dominance is commonly referred to as the preference for using one of our eyes when performing monocular tasks like looking through a small hole (e.g., camera, telescope, microscope; Coren and Kaplan, 1973). Most importantly, 66% of right-handers have a right sighting dominant eye (DE) (hereafter referred to as DE) and 34% a left DE (Bourassa et al., 1996). Therefore, if eye dominance has an influence on visual information interhemispheric transfer, then the broad consensus regarding the faster transfer of visual information from the right to the left hemisphere could stem from the over-representation of individuals with a right DE in a random population rather than being the fingerprint of a genuine brain lateralization.

In this light, the aim of the present study was to determine whether eye dominance does have an impact on IHTT for visual information exchange. Historically, the Poffenberger Paradigm (Poffenberger, 1912) has been considered as a choice method to evaluate the IHTT (Bashore, 1981; Marzi et al., 1991; Braun, 1992). This paradigm was built taking into account the crossed organization of the visual and motor systems: reaction time (RT) of button press in response to the onset of a lateralized visual target in an *uncrossed* condition (e.g., target on the right and responding hand on the right requiring no interhemispheric transfer) is subtracted to RT in a *crossed* condition (e.g., target on the left and responding hand on the right requiring an interhemispheric transfer). The difference obtained was considered as an evaluation of the IHTT, in our example from the right to the left hemisphere.

Electroencephalography (EEG) can also be used to estimate IHTT (Saron and Davidson, 1989). Indeed, following the presentation of a lateralized target, the visual areas contralateral to the stimulation will first be activated but this activation will be rapidly followed by the activation of the ipsilateral hemisphere. Then, given the excellent temporal resolution of the EEG, a precise evaluation of the IHTT can be obtained by comparing the latency of the visually evoked potential (VEP) recorded over each hemisphere.

To robustly determine whether eye dominance influences IHTT, we recorded VEPs while participants, with either a right or a left DE, were involved in the Poffenberger paradigm. Our goal was to gather two different evaluations (i.e., behavioral and electrophysiological) of IHTT to thoroughly assess the impact of eye dominance. Remarkably, the analyses of behavioral data (i.e., RTs) clearly revealed the pitfalls of using the Poffenberger paradigm for assessing IHTT. Specifically, the behavioral data does not allow assessing separately IHTT for each direction (from the right to the left and from the left to the right), precisely because of an eye dominance influence. Indeed, we observed that RT was shorter for stimuli presented in the visual hemifield contralateral to the DE, irrespectively of the responding hand side. Hence when considering one hand, the crossed-uncrossed difference is not only due to IHTT but rather to a combination between IHTT and this eye dominance influence. Such an equation with two unknowns renders impossible behavioral evaluation of IHTT for each direction. The only solution is then to average values obtained for each direction to cancel out the eye dominance influence meaning that, based on the behavior, only a global IHTT evaluation is possible (i.e., without considering the direction of the transfer). These results and their interpretation underlying the difficulties of using behavioral data were published separately (Chaumillon et al., 2014; see also Friedrich et al., 2017 concerning these difficulties). In the present article we used the electrophysiological data to estimate precisely the eye dominance influence on IHTT for each direction of information exchange.

## Materials and method

### Participants

The study was approved by the local ethics committee (CPP Sud—Méditerranée 1) and was performed in accordance with the ethical standards laid down in the Declaration of Helsinki (last modified, 2004). Forty participants, after giving written informed consent, performed a Poffenberger task while their EEG activity was recorded. All participants were healthy, reported normal or corrected-to-normal vision and showed no sign of neurological disorders. The results of 4 out of 40 participants (2 right-handers and 2 left-handers) were not included in the present study because of the poor quality of the EEG recordings which prevented to clearly identify the cortical response to the visual stimulations. The handedness of each participant was assessed by the Edinburgh Handedness Inventory (Oldfield, 1971; score = lateralization quotient). According to this test, a lateralization quotient of +100% represents extreme right hand preference and −100% extreme left hand preference. The mean lateralization quotient was 70.3% ±24.7 for right-handers (*n* = 22, mean age = 26.2 years ±5.4 (SD); 12 females) and −58.9% ± 23.8% for left-handers (*n* = 14, mean age = 23.2 years ± 4.4; 9 females).

The participants' eye dominance was assessed by the hole-in-card test (Miles, 1930) repeated three times. This test is known to be the most reliable to determine eye dominance (Taghavy and Kügler, 1987) and is not influenced by handedness. The rule was that if a given participant chose different eyes during these 3 assessments, he/she would not be included in the study. However, all participants showed consistent results across the three repetitions (i.e., the hole in the card was always aligned with the same eye). In each handedness group, this test allowed us to classify the participants in 2 sub-groups: right-handers with a left or a right DE (11 participants in each group) and left-handers with a left or a right DE (7 participants in each group).

### Experimental setup

In a dimly lit room, participants were comfortably seated on a chair in front of a table on which a response button was aligned with their body midline. Depending on the condition (see section Task, Protocol, and Stimulations), either their left or right index finger was resting on this button while the non-used hand was resting on the ipsilateral thigh. The participants were facing, at a viewing distance of 80 cm, a panel containing two lateralized white LEDs (74 cd/m^2^, 1.2° in visual angle), with their centers located at a horizontal distance of 10 cm (7.2° in visual angle) to the left and to the right of a smaller green central fixation LED (6 cd/m^2^; 0.4° in visual angle).

### Task, protocol, and stimulations

A trial started with the illumination of the fixation LED (viewed binocularly). Then, after a variable delay (i.e., 600–1,200 ms in 200 ms steps) either the left or the right target was presented for 100 ms. The participants had to press on the centrally placed button as quickly as possible after the LED illumination while keeping their gaze on the fixation LED (i.e., classical Poffenberger paradigm). Each participant performed 1,000 trials (i.e., 10 blocks of 100 trials) alternately with their left or right hand, the starting hand being balanced across participants. The inter-stimulus interval ranged between 1,400 and 2,500 ms and a short break was given to the participants between each experimental block. Among the 500 trials performed for each hand, 224 trials used the visual target that stimulated the left visual field (LVF) and 224 trials used the visual target that stimulated the right visual field (RVF) in a pseudo-randomly manner. In the remaining 52 trials, no target appeared. These “catch-trials,” pseudo-randomly distributed within the 5 blocks, helped preventing anticipation of target illumination. The experimental design therefore included four types of trials: left hand responses after LVF stimulations (LHand_LVF), or RVF stimulations (LHand_RVF) and right hand responses after LVF (RHand_LVF) or RVF (RHand_RVF) stimulations. To help participants maintain central fixation, the green LED remained lit throughout the trials.

### Electroencephalography data acquisition and pre-processing

**Figure 1:**
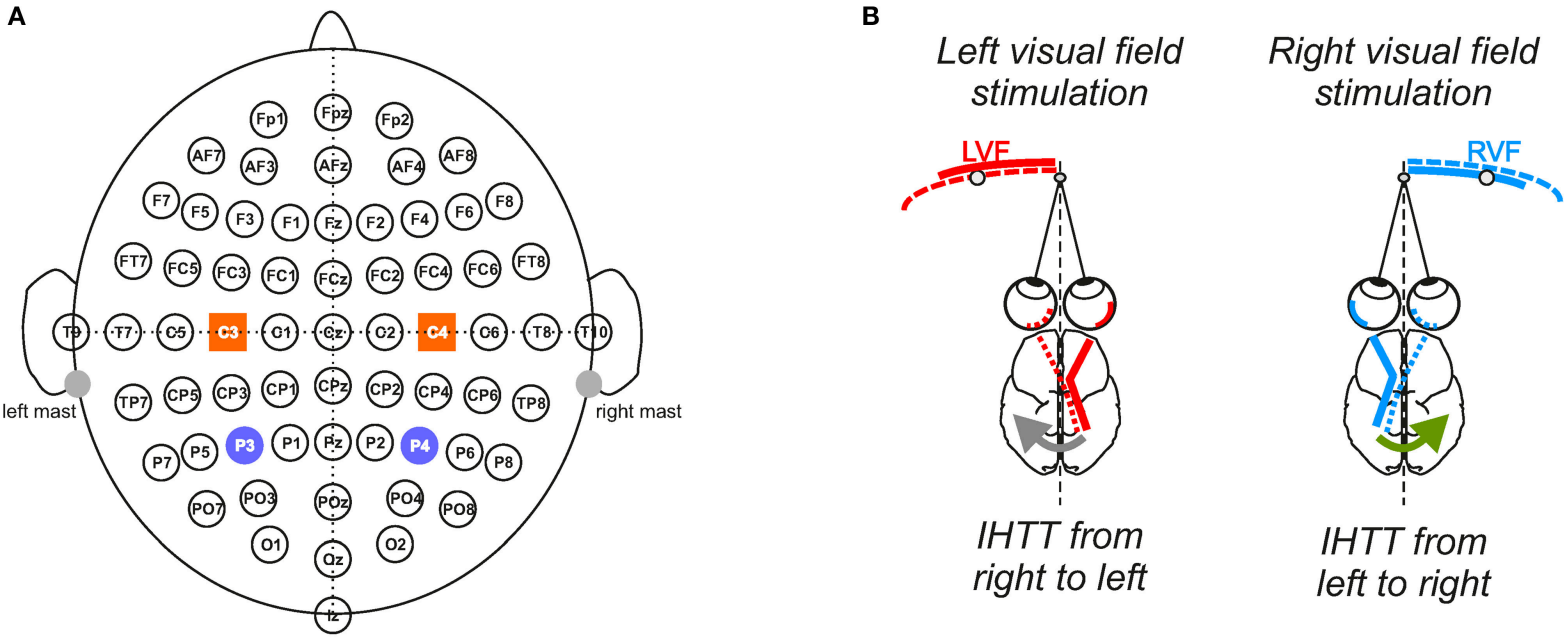
Experimental setup and conditions. **(A)** Electrophysiological recordings. Throughout the 10 blocks of 100 trials, electrophysiological recordings were performed from 64 electrodes in accordance with the extended 10/20 system. To analyze interhemispheric transfer time, we focused our analyses on the activities recorded over the posterior parietal (P3/P4) and central sites (C3/C4) depicted here as violet circles and orange squares, respectively. **(B)** Computation of the interhemispheric transfer time. Given the crossed organization of visual neural pathways, the direct response to the visual stimulation was recorded over the contralateral hemisphere whereas the indirect response, i.e., after interhemispheric transfer, was recorded over the ipsilateral hemisphere to the stimulation. Difference between the latencies of N160 peak (most negative deflection around 160 ms) recorded over both hemispheres gave an estimation of the interhemispheric transfer time (IHTT) from right to left (gray arrow) when the target appeared in LVF and from left to right (green arrow) when it appeared in RVF.

Electroencephalographic activity was recorded continuously from 64 pre-amplified Ag-AgCl electrodes (BioSemi ActiveTwo system; BioSemi, Amsterdam, The Netherlands) embedded on an elastic cap according to the standard 10–20 electrodes placement system (Sharbrough et al., 1991; Figure 1A). Eye movements and blinks were monitored by electro-oculography (EOG) using pairs of electrodes placed near both outer canthi and above and under the left orbit. The EEG and EOG signals were pre-amplified at the electrode sites, post-amplified with DC amplifiers, digitized at a sampling rate of 2,048 Hz and filtered online with a 0.16 Hz high-pass filter. The first pre-processing step was to reference the 68 channels (64 on the cap and 4 EOG channels) to the linked mastoids. Then signals were further bandpass-filtered offline (digital filters; 0.1–80 Hz; slope 24 dB/octave).

A first selection of trials was based on the behavior: trials associated with incorrect responses such as anticipation of target illumination (hand RT < 150 ms) or response omission (hand RT > 800 ms) were excluded (see Chaumillon et al., 2014). Then, a raw EEG data inspection was performed to also reject trials in which artifacts or eye movements (i.e., blinks and saccades) were detected. Across participants, a maximum of 5.1% of trials were rejected with all these criteria. EEG signals were then processed with an independent component analysis (ICA; Makeig et al., 1996; Jung et al., 2001) to further isolate and reduce remaining ocular artifacts (Hoffmann and Falkenstein, 2008).

In each group of participants, in order to directly investigate the two directions of interhemispheric transfer, we compared the cortical activations after LVF and RVF stimulations (i.e., IHTT from right to left and from left to right, respectively, see Figure 1B). Therefore, the LVF condition corresponds to the average of all LHand_LVF and RHand_LVF trials whereas the RVF condition corresponds to the average of all LHand_RVF and RHand_RVF trials. These averages across hands enabled us to double the number of valid epochs included in the average for each participant and each condition, resulting in a more accurate and a more reliable components detection. The number of included epochs per condition/participant did not differ significantly between the LVF and RVF conditions (*p* > 0.05; *t*-test, 382 epochs on average); suggesting similar signal-to-noise ratios of the VEPs averages between conditions. Finally, for each participant and condition, VEPs waveforms were obtained by averaging the EEG data of all the valid trials, considering epochs time-locked to stimulus onset (−200 to 400 ms) and the average amplitude of the 200 ms pre-stimulus period serving as baseline.

Then, we performed current source density (CSD) analyses (Stone and Freeman, 1971) using Laplacian transformation (Babiloni et al., 2001) with the software Brain Vision Analyzer (Brain Products GmbH, Munich, Germany). The signal was interpolated with a spherical spline interpolation procedure (Perrin et al., 1987, 1989) which involves the estimation of the second spatial derivation of the field potential (parameters: order of splines: 3; maximum degree of Legendre polynomials: 15; approximation parameter Lambda: 1.0e^−004^). CSDs are independent of the reference electrode site. Importantly, this method attenuates the detrimental effect of superimposition from multiple neural generators having different locations and orientations on the EEG recordings and therefore enhances their spatial resolution (see Kayser and Tenke, 2015; Vidal et al., 2015; for reviews). Moreover, through the enhancement of the EEG spatial resolution, CSD analyses also increase the temporal resolution of the recordings (Law et al., 1993). Accordingly, the use of CSD analyses allows one to measure more accurately the IHTT and also to disentangle the IHTT measured over posterior parietal and central sites. A supplementary low-pass filter (cut-off frequency set at 60 Hz) was performed on the CSD waves for graphical purposes only.

We focused our analyses on the activity recorded at P3/P4 and C3/C4 electrodes to study interhemispheric communication processes at the posterior parietal and central sites, respectively (Figure 1). Indeed, the P3 and P4 electrodes, which are positioned over the posterior parietal cortex (Koessler et al., 2009), are typically chosen to study the interhemispheric transfer of visual information (Pandya and Rosene, 1985; Pandya and Seltzer, 1986; Saron and Davidson, 1989). On the other hand, the C3/C4 electrodes allow investigating the transfer of information between both sensorimotor cortices (Ipata et al., 1997; Saron et al., 2003; Solodkin et al., 2004; Pfurtscheller et al., 2005). Corroborating previous studies (e.g., Di Russo et al., 2012), these posterior and central electrodes showed marked increased activities following the visual stimulations. As classically observed (e.g., Moes et al., 2007), the VEPs were composed of a positive peak (P100; also called P1) followed by a negative peak (N160, also called N1). We measured the latencies of these peaks and computed the amplitude of the P100-N160 from peak-to-peak. The N160 peak recorded over the posterior parietal cortex is known as an uncompounded indicator of IHTT (Brown and Jeeves, 1993; Ipata et al., 1997; Hausmann et al., 2013; Horowitz et al., 2014). Thus, the IHTT was computed using the posterior parietal electrodes (P3 and P4) by subtracting the latency of N160 recorded at the electrode contralateral to the stimulation (i.e., before interhemispheric transfer) from the N160 latency recorded at the ipsilateral electrode (i.e., after interhemispheric transfer). For each participant and each condition, the N160 peak was detected as the most negative deflection observed within the temporal window in which it was expected, i.e., between 120 and 210 ms (according to Moes et al., 2007). Peak detection was automatically detected and then visually verified and corrected when necessary. To be consistent and to allow the comparison between the transfer of information in posterior and central sites, we also used the N160 latencies to compute IHTT over C3/C4.

### Statistical analyses

All data were analyzed using R (R Development Core Team, 2017) with the R packages lme4 (Bates et al., 2015) and lsmeans (Lenth, 2016). Separately for right- and left-handers, we performed a series of linear mixed effects models to study the influence on IHTT of the factors DE (i.e., left or right) and Direction of interhemispheric transfer (i.e., from left to right or from right to left), considered as fixed effects. We entered intercepts for Participants as a random effect (Winter, 2013).

We also tested the effect of eye dominance on VEP amplitude. For each participant, we obtained 4 measures of P100-N160 amplitude issued from either the direct or the indirect activations (respectively recorded over the hemisphere contralateral and ipsilateral to the stimulation) for both visual field conditions (LVF, RVF). To focus on the effect of eye dominance on the direct/indirect activation pattern, we pooled responses for both visual fields. In this case we performed linear mixed effects models with DE (i.e., left or right) and Activation type (i.e., direct or indirect) as fixed effects and intercepts for Participants as a random effect.

For both IHTT and VEP amplitude analyses, we computed the 95% confidence intervals for the difference between values for both directions and between values for both activation types. Differences were always computed by subtracting values of the group with the smaller mean from values of the group with the larger mean.

To specifically investigate the potential effect of the method used to compute the VEPs, we compared IHTT based on CSD and bipolar analyses in right-handers with a linear mixed effects analysis. DE (i.e., left or right) and Method (CSD or bipolar) were entered as fixed effects and intercepts for Participants were entered as random effect. Finally to compare IHTT values between P3/P4 and C3/C4 electrodes we performed a linear mixed effects model with the factors DE (i.e., left or right) and Electrodes sites (P3/P4 or C3/C4) as fixed effects and intercepts for Participants as random effect.

In all cases, *P*-values were obtained by likelihood ratio tests of the full model with the considered effect against the model without that effect.

## Results

### Right-handers

**Figure 2:**
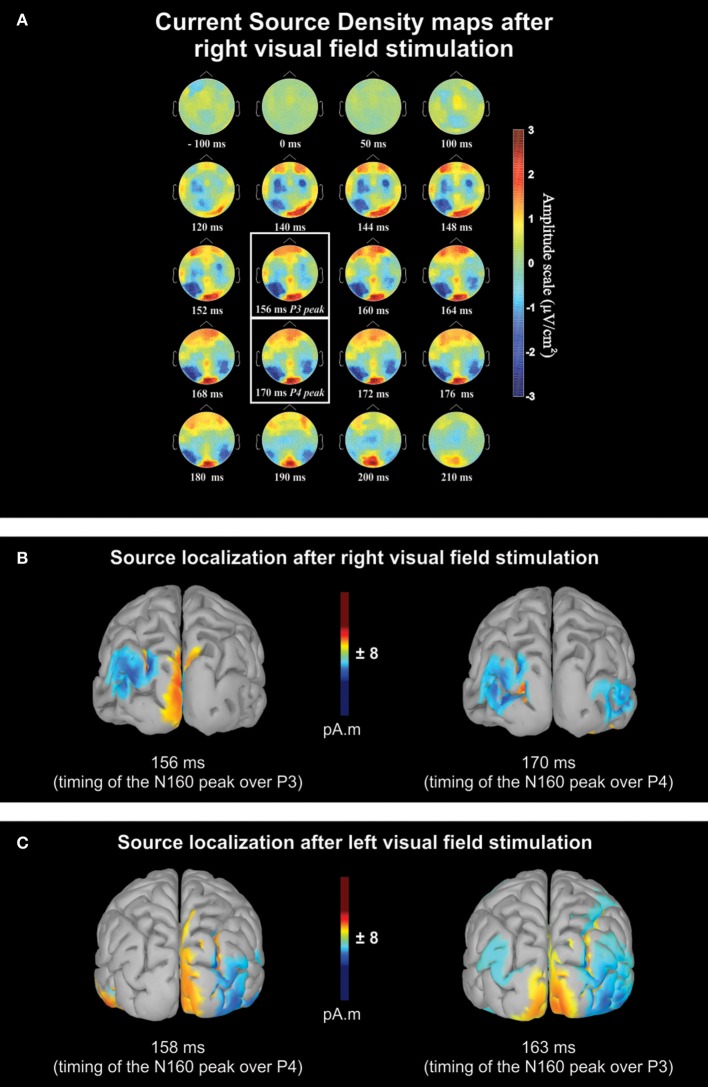
Qualitative overview based on the averaged data of the 11 right-handers with a right dominant eye. **(A)** Sequential current source density (CSD) maps from 100 ms before to 210 ms after right visual field stimulation. The maps are presented for different times with closer intervals (between 140 and 180 ms) for the period where peak negativity (N160) was expected. The two white boxes contain the maps recorded at mean latency of the maximum negative deflection recorded over P3 and P4 electrodes. Blue and red areas on the maps correspond to negative and positive voltage, respectively. Note that the activation of the contralateral hemisphere with respect to the stimulation precedes the ipsilateral hemisphere activation by a few milliseconds. Activations over sensori-motor cortices (C3 and C4 electrodes) occurring earlier than posterior N160 are thought to be related to visuo-motor integration. **(B)** Estimated source maps on the cortical surface (LORETA) at the N160 latency based on the grand average after right visual field (RVF) stimulations and **(C)** after left visual field (LVF) stimulations. In both conditions, source localization shows an interhemispheric transfer ~160–170 ms, after the visual stimulation, occurring in the medium/superior occipital gyrus (Brodmann area 19). For the sake of clarity, only activity sources that were 11% above minimal activation are shown.

Figure 2A shows the scalp topography of Current Source Density (CSD) based on the average of the 11 right-handers with a right DE at different times before and after a RVF stimulation. The maps reveal a large negativity that developed over the left hemisphere after the visual stimulation. This negativity peaked ~160 ms post-stimulation and was more pronounced over posterior sites. The maps also show a negative wave expanding over the right posterior hemisphere but which lagged the negativity observed in the left hemisphere. This lag represents the time required to transfer the information from the left to the right hemisphere (see below “Posterior sites”). Clear negativities also developed over the sensorimotor cortices but with shorter latencies than for the posterior parietal regions. These last activations are known to be linked to visuo-motor integration (Berlucchi, 1972; Milner and Lines, 1982; Rugg et al., 1984; Saron et al., 2003; see below “Central sites”).

Figures 2B,C show the estimated neural source of the N160 recorded over the posterior parietal regions after a right and left visual stimulation, respectively (the time-matched CSD maps of Figure 2B are indicated with white boxes in Figure 2A). These estimations were obtained through low-resolution brain electromagnetic tomography (LORETA; Pascual-Marqui et al., 1994), implemented in Brainstorm software (Tadel et al., 2011; http://neuroimage.usc.edu/brainstorm). The sources reconstruction were based on the waves obtained from the binocular recordings of the same participants as in Figure 2A. We employed the minimum-norm technique implemented in Brainstorm software to resolve the inverse problem and estimate the VEP cortical sources of the left and right hemispheres (i.e., in cases with and without interhemispheric transfer). We imported the data from all sensors processed and averaged for each condition and electrode. The forward model was computed for each condition using a symmetric boundary element method (BEM, Gramfort et al., 2010) on the anatomical MRI Colin 27 brain template, a predominant volume conductor model from the Montreal Neurological Institute (Mosher et al., 1999; Huang et al., 2016). The cortical sources were searched at N160 peaks determined from the waveforms of the grand average. They suggest that N160 activation recorded over P3 and P4 were generated approximately in medium-superior occipital gyrus (BA 19; MNI template; Evans et al., 1992a,b). This is in agreement with previous studies suggesting that the main part of callosal transfer of visual information occurs in extrastriate areas (Pandya and Rosene, 1985; Pandya and Seltzer, 1986). The estimated neural source computed from the 3 other groups for each condition (i.e., LVF or RVF stimulations) are reported in Supplementary Figures 1–3. These source reconstructions all showed activations in the medium-superior occipital gyrus (BA 19) region suggesting that the neural processes underlying the N160 peaks were similar across all groups.

#### Posterior sites

##### Interhemispheric transfer time

**Figure 3:**
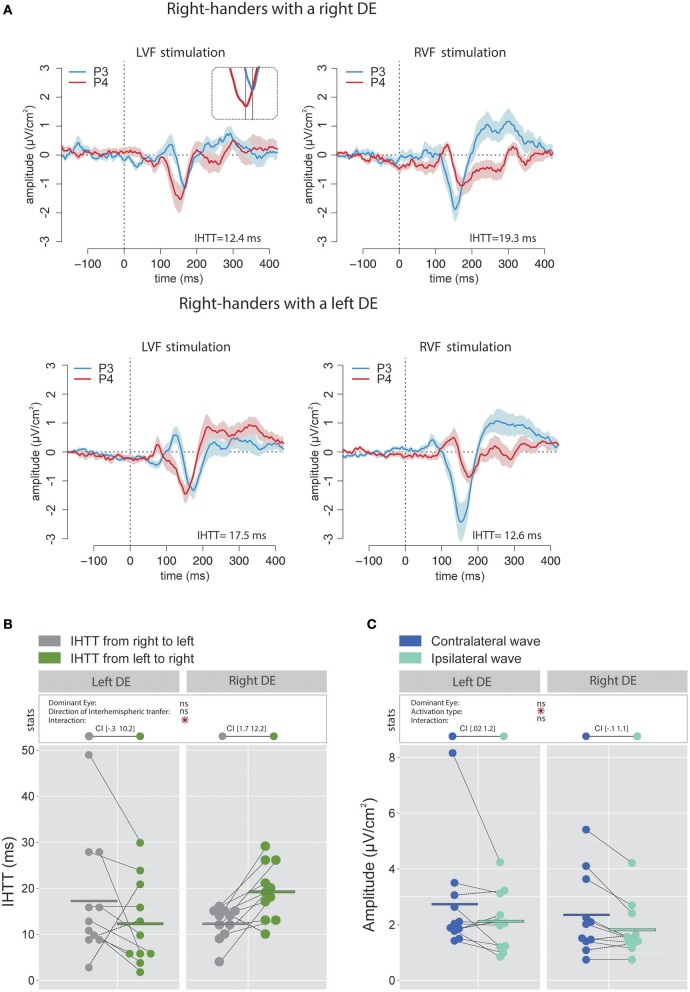
Waveforms and average results computed over posterior sites after Laplacian transformation in right-handers. **(A)** Grand average CSD waveforms recorded over P3 (blue waves) and P4 (red waves) electrodes in the two experimental conditions (LVF or RVF stimulation). Shading blue and red areas represent the SEM across subjects. **(B)** Individual data and mean IHTT as a function of the DE and of the direction of interhemispheric transfer. Statistical analysis showed that the asymmetry in IHTT was dependant on the side of the DE: faster from the right to the left (gray circles) for right-handers with a right DE and faster in the reverse direction (green circles) for right-handers with a left DE. The values between brackets represent the 95% Confident Intervals of difference between both directions, computed by subtracting values of the group with the smaller mean from values of the group with the larger mean. **(C)** Individual data and mean P100-N160 amplitudes recorded over contralateral (dark blue circles) and ipsilateral (light blue circles) hemispheres to the stimulation. The hemisphere contralateral to the stimulation showed greater P100-N160 amplitudes than the ipsilateral hemisphere. Asterisks show a significant effect (*p* < 0.05).

Figure 3A contains the grand average of CSD waveforms recorded over electrodes P3 and P4 in response to LVF and RVF stimulations in right-handers with either a right (top) or left (bottom) DE. The peak negativity (N160) occurred first in the hemisphere contralateral to the stimulated visual hemifield and with a slight temporal delay in the ipsilateral hemisphere. Figure 3B shows means and individual values of IHTT for each direction in the two groups of right-handers (i.e., with a left DE and with a right DE). Statistical analyses did not reveal a main effect of the factor DE [$\chi_{\left(1\right)}^{2}$ = 0.07, *p* = 0.79] nor of the factor Direction of interhemispheric transfer [$\chi_{\left(1\right)}^{2}$ = 0.21, *p* = 0.65] but revealed a significant interaction between these two factors [$\chi_{\left(1\right)}^{2}$ = 8.85, *p* = 0.003]. This interaction indicates that the asymmetry in IHTT was strongly dependent on the DE. Indeed, right-handers with a right DE showed an interhemispheric transfer that was faster by [1.7 12.2] ms from right to left than from left to right (95% CI). Importantly, in opposition to current models of brain asymmetries, right-handers with a left DE showed faster interhemispheric transfer by [−0.3 10.2] ms from the left to right than from right to left.

**Figure 4:**
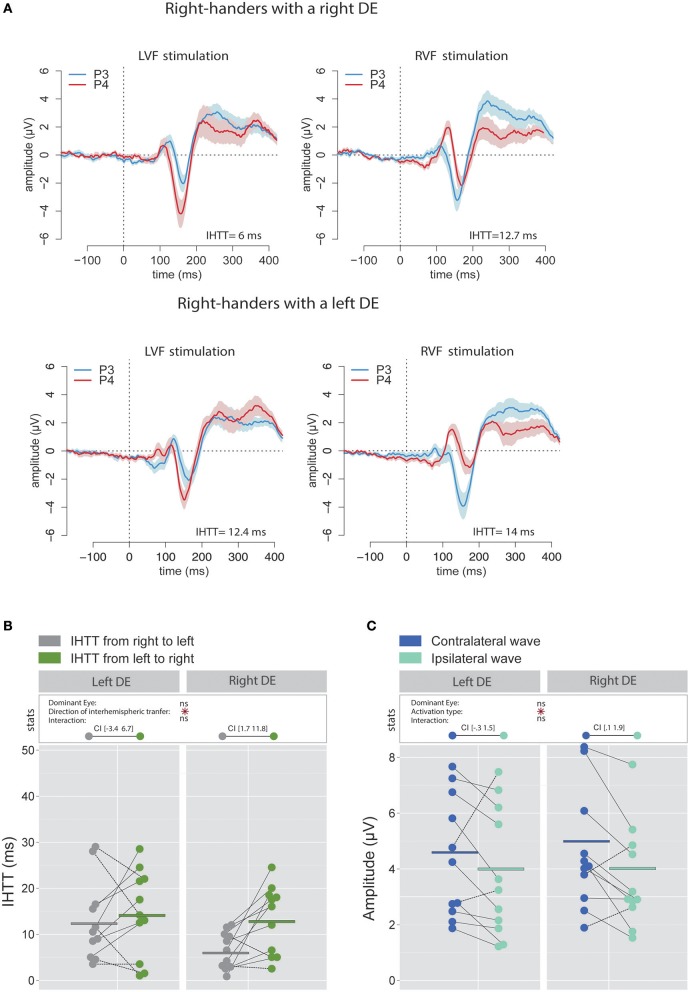
Waveforms and average results from bipolar recordings computed over posterior sites. **(A)** Grand average bipolar waveforms recorded over P3 (blue waves) and P4 (red waves) electrodes in the two experimental conditions (LVF or RVF stimulation). Shading blue and red areas represent the SEM across subjects. **(B)** Individual data and mean IHTT as a function of the DE and of the direction of interhemispheric transfer. Statistical analysis showed a main effect of the direction of the interhemispheric transfer: IHTT was faster from right to left (gray circles) than from left to right (green circles). Nevertheless, as revealed by the 95% Confident Intervals reported between brackets, this pattern was only significant in right-handers with a right DE. **(C)** Individual data and mean P100-N160 amplitudes recorded over contralateral (dark blue circles) and ipsilateral (light blue circles) hemispheres to the stimulation. The hemisphere contralateral to the stimulation showed greater P100-N160 amplitudes than the ipsilateral hemisphere. Asterisks show a significant effect (*p* < 0.05).

To allow comparison with previous studies that did not use the CSD analyses (e.g., Rugg et al., 1984, 1985; Brown and Jeeves, 1993), we also report, for right-handers, the IHTT computed from bipolar analyses (see Figures 4A,B). In agreement with Saron et al. (2003) who also reported both measures, IHTT were shorter when computed over bipolar (mean IHTT across all right-handers and conditions = 11.3 ± 8.1 ms) than over CSD (15.4 ± 8.9 ms) waveforms [$\chi_{\left(1\right)}^{2}$ = 7.16, *p* = 0.008]. Using the same linear mixed effects model as for the CSD analyses above, we found a significant effect of the factor Direction of interhemispheric transfer [($\chi_{\left(1\right)}^{2}$ = 4.78, *p* = 0.029] which showed that the transfer was faster from right to left (9.2 ± 7.4 ms) than from left to right (13.4 ± 8.3 ms). Nevertheless, when considered separately for each DE, this IHTT asymmetry was actually present only in right-handers with a right DE: the interhemispheric transfer was faster by [1.7 11.8] ms from right to left than from left to right. By contrast, the asymmetry was not present in right-handers with a left DE: the difference in IHTT from right to left minus IHTT from left to right was of [−3.4 6.7] ms.

Based on these observations, we suggest that the classical result of IHTT asymmetry (i.e., faster from right to left, see Introduction) was obtained because of the greater number of participants with a right DE in a random right-handed population (Bourassa et al., 1996) and of the use of bipolar analyses of the EEG recordings in previous studies (Saron and Davidson, 1989; Brown et al., 1994, 1998; Endrass et al., 2002; Barnett and Kirk, 2005; Barnett et al., 2005; Moes et al., 2007; Patston et al., 2007; Iwabuchi and Kirk, 2009) which have smaller spatial resolution than CSDs (see Discussion for details).

##### P100-N160 amplitude

The linear mixed effects analysis performed on the P100-N160 amplitude (CSD data; Figure 3C) revealed a significant effect of Activation type (i.e., direct or indirect) which indicated that the amplitude of the contralateral wave (2.53 ± 1.67 μV/cm^2^) was larger than the ipsilateral wave [1.97 ± 1.01 μV/cm^2^; $\chi_{\left(1\right)}^{2}$ = 6.81, *p* = 0.01]. No significant influence of the factor DE was observed [$\chi_{\left(1\right)}^{2}$ = 0.41, *p* = 0.52].

Similarly, when performing the analyses on the P100-N160 amplitude computed using the bipolar waveforms (see Figure 4C), the linear mixed effects analysis also revealed that the VEP was significantly larger [$\chi_{\left(1\right)}^{2}$ = 5.69, *p* = 0.017] when recorded over the hemisphere contralateral to the stimulation (4.8 ± 2.8 μV) than over the ipsilateral hemisphere (4.02 ± 2.5 μV).

#### Central sites

##### Interhemispheric transfer time

**Figure 5:**
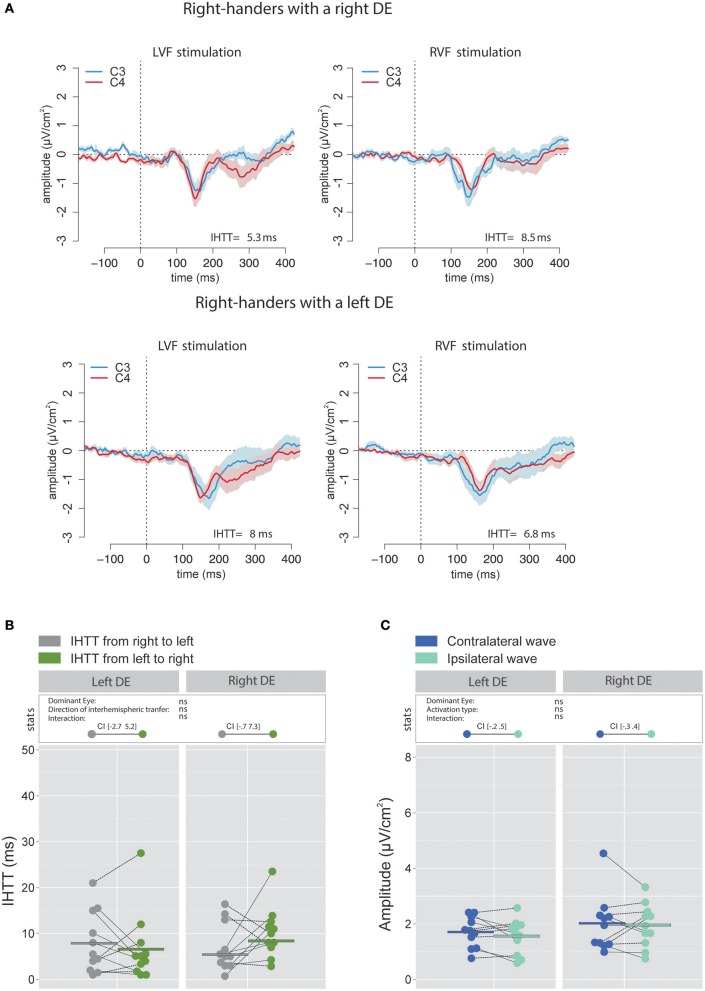
Waveforms and average results computed over central sites after Laplacian transformation in right-handers. **(A)** Grand average CSD waveforms recorded over C3 (blue waves) and C4 (red waves) electrodes in the two experimental conditions (LVF or RVF stimulation). Shading blue and red areas represent the SEM across subjects. **(B)** Individual data and mean IHTT as a function of the DE and of the direction of interhemispheric transfer. Contrary to what we observed over posterior sites, there was no significant influence of the DE and of the direction of the interhemispheric transfer [i.e., from right to left (gray circles) or from left to right (green circles)] on IHTT. **(C)** Individual data and mean P100-N160 amplitudes recorded over contralateral (dark blue circles) and ipsilateral (light blue circles) hemispheres to the stimulation. The amplitude of the P100-N160 recorded over both hemispheres did not significantly differ.

The analyses performed on the signal recorded over the electrodes C3 and C4 revealed that, in accordance with previous works (e.g., Saron et al., 2003), IHTT were shorter when computed over central (global mean 7.1 ± 7.45 ms) than over posterior (global mean 15.4 ± 8.9 ms) sites [$\chi_{\left(1\right)}^{2}$ = 21.99, *p* < 0.001]. More importantly, the linear mixed effects analysis performed to test the influence of the factors DE and Direction of interhemispheric transfer did not reveal significant effects of DE [$\chi_{\left(1\right)}^{2}$ = 0.31, *p* = 0.58] or Direction of interhemispheric transfer [$\chi_{\left(1\right)}^{2}$ = 0.51, *p* = 0.48] on the central IHTT and no significant interaction between these two factors [$\chi_{\left(1\right)}^{2}$ = 2.66, *p* = 0.103; see Figures 5A,B).

On the contrary, analyses conducted with bipolar waveforms showed a significant effect of the factor Direction of interhemispheric transfer [$\chi_{\left(1\right)}^{2}$ = 4.11, *p* = 0.043] corresponding to a faster transfer from right to left. This observed faster interhemispheric transfer for the right to left direction echoed the results obtained with electrodes overlying the posterior parietal sites (see Ipata et al., 1997; for a similar result). A plausible interpretation is that when analyzed with bipolar waveforms, activities over central sites may largely reflect more posterior activities through volume conduction. CSD analyses strongly limit volume conduction, and hence more accurately render the cortical activity under the surface electrodes (Kayser and Tenke, 2015; Vidal et al., 2015). Nevertheless, it should be noted that a supplementary linear mixed effects analysis with three fixed effects (DE, Site and Direction of interhemispheric transfer) performed on CSD values did not show a significant interaction between the factors Direction of interhemispheric transfer and Site [$\chi_{\left(1\right)}^{2}$ < 0.001, *p* = 0.99]. This may indicate that even with CSD analyses, neural activities from posterior sources could still slightly influence the signals recorded on central electrodes.

##### P100-N160 amplitude

Statistical analysis did not reveal significant influence of the factor DE [$\chi_{\left(1\right)}^{2}$ = 1.34, *p* = 0.25] or Activation type [$\chi_{\left(1\right)}^{2}$ = 0.86, *p* = 0.35] and no significant interaction between these two factors [$\chi_{\left(1\right)}^{2}$ = 0.20, *p* = 0.66] on the P100-N160 amplitude, as computed with CSD waveforms (Figure 5C).

Similarly to those performed on IHTT, the analyses conducted on the P100-N160 amplitude computed with bipolar waveforms recorded over central sites revealed a pattern similar to the one obtained over posterior sites: the amplitude of the P100-N160 wave was significantly larger [$\chi_{\left(1\right)}^{2}$ = 21.51, *p* < 0.001] when recorded over the hemisphere contralateral (4.99 ± 2.29 μV) than over the hemisphere ipsilateral (3.92 ± 2.6 μV) to the stimulation. This result is also in agreement with the hypothesis raised above of a large effect of volume conduction on bipolar recordings.

### Left-handers

#### Posterior sites

##### Interhemispheric transfer time

**Figure 6:**
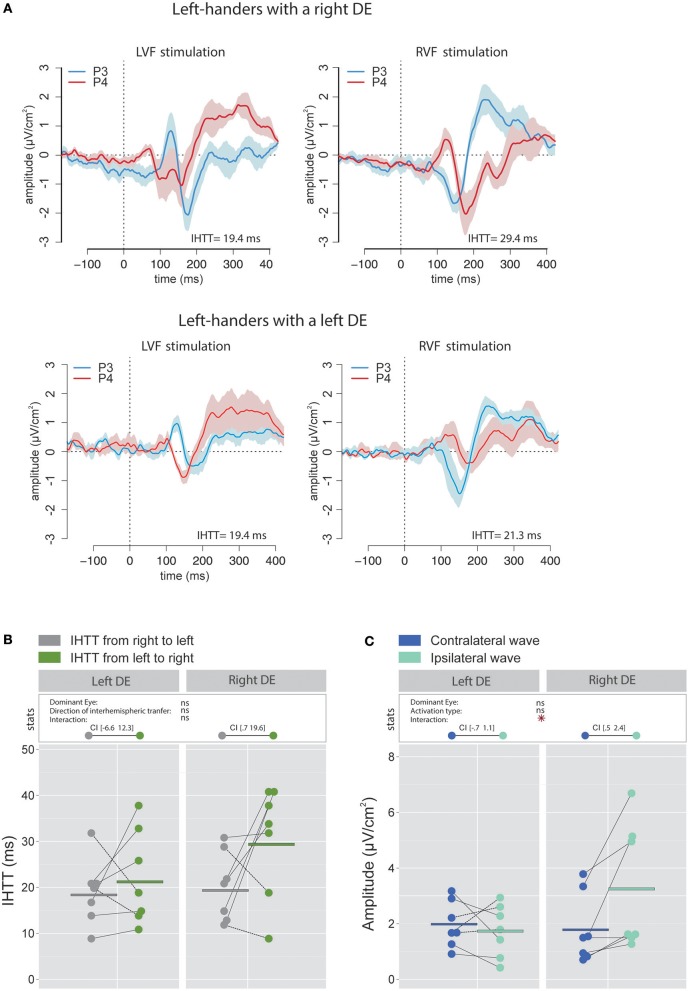
Waveforms and average results computed over posterior sites after Laplacian transformation in left-handers. **(A)** Grand average CSD waveforms recorded over P3 (blue waves) and P4 (red waves) electrodes in the two experimental conditions (LVF or RVF stimulation). Shading blue and red areas represent the SEM across subjects. **(B)** Individual data and mean IHTT as a function of the DE and the direction of interhemispheric transfer. Statistical analysis revealed a strong tendency toward an effect of the factor Direction of interhemispheric transfer (*p* = 0.06). When considering each group of eye dominance separately, it appears that this faster IHTT from right to left (gray circles) than from left to right (green circles) was significant only in left-handers with a left DE. The values between brackets represent the 95% Confident Intervals of difference between both directions. **(C)** Individual data and mean P100-N160 amplitudes recorded over contralateral and ipsilateral hemispheres to the stimulation. Contrary to the classical pattern, left-handers with a right DE showed larger P100-N160 amplitude over ispilateral (i.e., after the interhemispheric transfer, light blue circles) than contralateral (dark blue circles) hemisphere with respect to the stimulation. Asterisks show a significant effect (*p* < 0.05).

The linear mixed effects analysis performed using IHTT computed with CSD waveforms indicated that the interhemispheric transfer from right to left tended to be faster than the interhemispheric transfer from left to right (the effect of the factor Direction of interhemispheric transfer was close to significance level [$\chi_{\left(1\right)}^{2}$ = 3.51, *p* = 0.061; Figures 6A,B). This result may largely be due to the difference observed in participants with a right DE. Indeed, left-handers with a right DE showed an interhemispheric transfer that was faster by [0.7 19.6] ms from right to left than from left to right (95% CI). On the contrary, the asymmetry was not present in left-handers with a left DE: the difference in IHTT from right to left minus IHTT from left to right was of [−6.6 12.3] ms.

##### P100-N160 amplitude

Contrary to our expectation, the statistical analysis revealed a significant interaction between factors Activation type and DE [$\chi_{\left(1\right)}^{2}$ = 6.11, *p* = 0.014] and no main effect of these factors. Unexpectedly, VEPs recorded in participants with a right DE were greater in the hemisphere ipsilateral to the simulation than in the hemisphere contralateral to the stimulation with a difference of [0.5 2.4] μV/cm^2^ (Figure 6C). Conversely, no significant difference was observed between the VEP amplitude recorded over both hemispheres for participants with a left DE: the difference between amplitude in the contralateral hemisphere minus amplitude in the ipsilateral hemisphere was of [−0.7 1.1] μV/cm^2^. Hence, left-handers with a right DE showed larger indirect (i.e., after interhemispheric transfer) than direct wave amplitudes, in contradiction to the classical pattern reported in the literature (e.g., Brown et al., 1994; Moes et al., 2007) and the results that we obtained in right-handers. However, this finding is consistent with, and then reinforces, the view of distinct sensorimotor circuits of left-handers with a right DE (Petit et al., 2014; see Discussion).

#### Central sites

##### Interhemispheric transfer time

**Figure 7:**
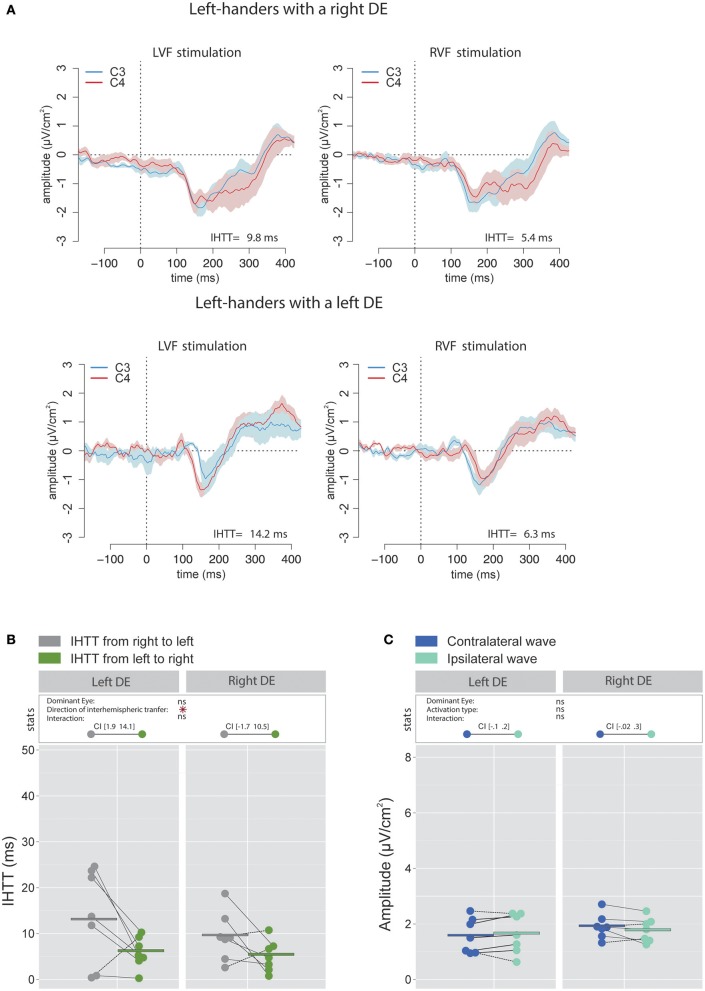
Waveforms and average results computed over central sites after Laplacian transformation in left-handers. **(A)** Grand average CSD waveforms recorded over C3 (blue waves) and C4 (red waves) electrodes in the two experimental conditions (LVF or RVF stimulation). Shading blue and red areas represent the SEM across subjects. **(B)** Individual data and mean IHTT as a function of the DE and the direction of interhemispheric transfer. The statistical analysis revealed a main effect of the factor Direction of interhemispheric transfer indicating a faster interhemispheric transfer from left to right hemisphere (green circles) than from right to left hemisphere (gray circles). Nevertheless, as revealed by the 95% Confidence Intervals reported between brackets, the difference between the two directions was statistically significant only for Left-handers with a left DE. **(C)** Individual data and mean P100-N160 amplitudes recorded over contralateral (dark blue circles) and ipsilateral (light blue circles) hemispheres to the stimulation. The amplitudes of contralateral and ipsilateral P100-N160 complexes were similar in both left-handers with a left or a right DE. Asterisks show a significant effect (*p* < 0.05).

As in right-handers, the IHTT was shorter over central sites than over posterior sites [global means: 8.9 ± 6.2 ms vs. 22.2 ± 9.3 ms; $\chi_{\left(1\right)}^{2}$ = 27.4, *p* < 0.001]. Concerning central sites, the statistical analysis revealed a main effect of the factor Direction of interhemispheric transfer [$\chi_{\left(1\right)}^{2}$ = 7.06, *p* = 0.008] indicating a faster interhemispheric transfer from left to right hemisphere (5.8 ± 3.71 ms) than from right to left hemisphere (12 ± 8.6 ms; see Figures 7A,B). Nevertheless, when considered separately for each group of DE, the difference between the two directions was statistically significant for left-handers with a left DE with values of [1.9 14.1] ms, but not for left-handers with a right DE [(−1.7 10.5)ms].

##### P100-N160 amplitude

There was no significant impact of the factor DE [$\chi_{\left(1\right)}^{2}$ = 0.42, *p* = 0.52] nor of the factor Activation type [$\chi_{\left(1\right)}^{2}$ = 0.44, *p* = 0.51] on the amplitude of the P100-N160 complex. The interaction between these two factors did not reach a significant level [$\chi_{\left(1\right)}^{2}$ = 3.18, *p* = 0.08; see Figure 7C].

## Discussion

**Figure 8:**
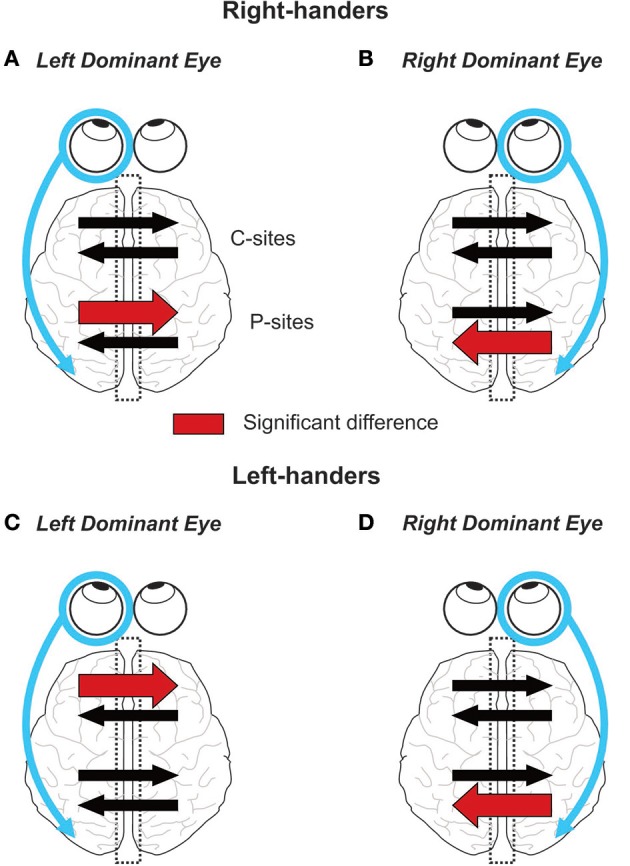
Graphical summary of the results. On each panel, the encircled eye indicates the DE and the connected arrow its preferential relationship with its ipsilateral hemisphere (Shima et al., 2010; Chaumillon et al., 2014). Concerning the IHTT over the posterior sites (P-sites), the present experiment revealed that in right-handers, the asymmetry in IHTT strictly depends on the eye dominance. Indeed, right-handers with a left DE **(A)** show faster interhemispheric transfer from left to right than from right to left whereas right-handers with a right DE **(B)** show the opposite pattern. In left-handers, individuals with a left DE **(C)** show no asymmetry whereas individuals with a right DE **(D)** show a faster interhemispheric transfer from right to left than from left to right. Concerning the IHTT over central sites (C-sites), right-handers show no difference between the two interhemispheric transfer directions. On the contrary, left-handers with a left DE show faster IHTT from left to right whereas left-handers with a right DE show no difference between the two interhemispheric transfer directions.

Communication between the cerebral hemispheres is paramount to several brain processes, notably those underlying visual perception and sensorimotor transformation. Using the visual-evoked potential (VEP) technique, the present study revealed that the lateralization of the visual system, referred to as eye dominance, has a strong impact on the interhemispheric transfer of visual information. Indeed, in right-handers we found a marked asymmetry in IHTT at the level of the posterior parietal cortices that depended on the side of the DE: right-handers with a right DE showed faster interhemispheric transfer from right to left than from left to right whereas right-handers with a left DE showed the opposite asymmetry. In other words, interhemispheric transfer was always faster from the ipsilateral to the contralateral hemisphere with respect to the DE. To the best of the authors' knowledge, these results provide the first demonstration of an influence of the eye dominance on the communication between the two posterior parietal cortices. In left-handers, the effect of eye dominance on posterior sites communication was less straightforward: an IHTT asymmetry with a faster interhemispheric transfer from right to left was observed only in left-handers with a right DE. Considering both groups of handedness, the interhemispheric transfer occurring at the central region of the corpus callosum was only slightly influenced by eye dominance: an asymmetry was exclusively found in left-handers with a left DE with a quicker transfer from the left to the right hemisphere (see Figure 8 for a graphical summary of the results based on Current Source Density (CSD) analyses).

### Right-handers

As indicated in the Introduction, the interhemispheric transfer of visual information in right-handers has been classically considered as being faster from the right to the left hemisphere. The present findings strongly suggest that the large consensus regarding the direction of this asymmetry (e.g., Saron and Davidson, 1989; Brown et al., 1994, 1998; Endrass et al., 2002; Barnett and Kirk, 2005; Barnett et al., 2005; Moes et al., 2007; Patston et al., 2007; Iwabuchi and Kirk, 2009; but see Nowicka et al., 1996; Horowitz et al., 2014) arose from the fact that previous studies have not taken into account the participants' eye dominance. Indeed, by not considering eye dominance, the percentage of right-handed participants with a right DE in previous studies might have approximated 66% (see Introduction and Bourassa et al., 1996). Our results showed that these participants, contrary to those with a left DE (i.e., ~34% of a random right-handed population), showed faster IHTT from the right to the left hemisphere. Averaging the IHTT of a random population therefore biases the results toward those pertaining to the participants with a right DE, most likely leading to the significant faster right to left hemisphere transfer reported by previous studies.

Concerning the P100-N160 amplitude recorded over posterior sites, the present results are in agreement with the classical reported pattern of larger waves over the contralateral hemisphere with respect to the stimulation than over the ipsilateral hemisphere (Rugg et al., 1984, 1985; Brown and Jeeves, 1993; Saron et al., 2003) regardless of the DE.

From a methodological perspective, the present study demonstrated the importance of applying a spatial filter to the EEG data when investigating interhemispheric communication. Indeed, the quicker interhemispheric transfer from left to right than from right to left in right-handers with a left DE was only revealed when applying a CSD transform. Moreover, our CSD recordings did not show the faster interhemispheric transfer from the right to the left hemisphere that has been observed by Ipata et al. (1997) over central sites with bipolar recordings. It should be noted, however, that when using bipolar recordings to compute this IHTT, we found the same asymmetry as in Ipata et al.'s (1997). CSD transform is a method that allows separating brain activities in both the spatial (Nuñez, 1981; Babiloni et al., 2001; Kayser and Tenke, 2015) and temporal (Burle et al., 2015) domains. Consequently, CSDs are much less affected by volume conduction and far-field generators than bipolar recordings (Manahilov et al., 1992; Nuñez et al., 1994; Tenke and Kayser, 2012). Hence, the asymmetry in the IHTT observed here and by Ipata et al. (1997) at the C3 and C4 electrodes with bipolar recordings is likely to be the mere echo of the asymmetry phenomenon occurring at posterior sites.

As reported in several studies, the response to the visual stimuli (N160 latency) had shorter latencies in central sites than in parietal sites (see Berlucchi, 1972; Milner and Lines, 1982; Lines et al., 1984; Rugg et al., 1984; Brown et al., 1994; Ipata et al., 1997; Saron et al., 2003; Barnett and Corballis, 2005). Fast visuo-motor channels, including the direct projections from parieto-occipital areas (also known as V6A) to the dorsal premotor cortex (Wise et al., 1997), could be responsible for these early central activations.

Importantly, the absence of IHTT asymmetry at central sites in right-handers after EEG spatial filtering sheds new light on the classical Poffenberger paradigm. Indeed, numerous pieces of evidence indicate that the transfer of information for this manual task occurs at central sites: behavioral estimations of IHTT are similar to EEG values obtained at central sites (Lines et al., 1984; Rugg et al., 1984; Saron and Davidson, 1989; Nowicka et al., 1996; Ipata et al., 1997; Saron et al., 2003), fMRI studies showed that, when required for the behavior, the interhemispheric transfer activates the genu of the corpus callosum (Tettamanti et al., 2002; Weber et al., 2005), an interhemispheric transfer occurring at posterior sites would imply a sensitivity of behavioral IHTT estimations to visual stimulation parameters, which is not the case (Berlucchi et al., 1971, 1977; Milner and Lines, 1982; Lines et al., 1984). Then the absence of asymmetry at the central level in the present study implies that no asymmetry should be observed in the Poffenberger paradigm. This prediction reinforces the conclusion we have reached recently using Monte-Carlo simulations (Chaumillon et al., 2014). The results of these simulations supported the hypothesis that the classical result of asymmetry in behavioral experiments (i.e., Poffenberger paradigm, Marzi et al., 1991; Marzi, 2010) emerged from the merging of results of right-handers with a right and a left DE which are not equally represented in a random population of right-handers (Bourassa et al., 1996).

### Left-handers

Left-handers with a right DE showed an asymmetrical IHTT at posterior sites, with faster interhemispheric transfer from the right to left hemisphere. This finding contrasts with the lack of asymmetry reported in previous EEG studies that measured IHTT in left-handers (Savage and Thomas, 1993; Iwabuchi and Kirk, 2009). Again, these previous studies did not take into account the eye dominance. The fact that a majority of left-handers have a left DE (60% according to Bourassa et al., 1996) could have biased the results of previous studies toward those that we found for these participants, i.e., an absence of asymmetry. Indeed, in the present study, the side of the stimulated visual field had no significant effect on the IHTT in left-handers with a left DE. Our findings therefore suggest that the influence of eye dominance on the IHTT is weaker for the left-handers than for the right-handers. Nevertheless, it should be noted that the relatively small sample of participants tested here (i.e., *n* = 7 for both groups of left-handers) calls for further investigations in this population.

Taking into account the eye dominance allowed us to pinpoint another peculiarity of the VEPs of left-handers with a right DE. Indeed, contrary to all other groups of participants and to what is classically reported in the literature (e.g., Rugg et al., 1984, 1985; Brown and Jeeves, 1993; Saron et al., 2003), these individuals showed larger posterior parietal P100-N160 amplitude after interhemispheric transfer than before interhemispheric transfer. To our knowledge, this pattern of activation has never been reported. Although the nature of this phenomenon cannot be elucidated here, this novel finding may be related to the specificity of the visuo-attentional network in these individuals (Azémar, 2003; Azémar et al., 2008; Petit et al., 2014).

For the central sites, the statistical analyses revealed an asymmetrical communication only in left-handers with a left DE, with a faster interhemispheric transfer from the left to the right hemisphere than in the reverse direction. This population was also the only one among the four tested populations that did not show a faster IHTT over posterior parietal sites, from the ipsilateral to the contralateral hemisphere with respect to the DE. This singular pattern again argues for the existence of peculiar interhemispheric processes in left-handed people (e.g., Cherbuin and Brinkman, 2006).

### Neurophysiological considerations

The interhemispheric transfer of information relies heavily on the corpus callosum which is the largest commissure in the brain (Gazzaniga, 2000; Aboitiz and Montiel, 2003; Fabri et al., 2014). As a possible neurophysiological substrate for the generalization of a faster IHTT from the right to the left hemisphere made in previous studies, Marzi et al. (1991) proposed that a larger number of fibers could connect the right hemisphere to the left one, than in the reverse direction. This hypothesis has been supported by the work of Putnam et al. (2010) showing that in the splenium, which is critical for interhemispheric communication between visual areas (Pandya et al., 1971; Gazzaniga, 2000; Aboitiz and Montiel, 2003), a larger number of fibers cross from the right to the left hemisphere than in the reverse direction. Nevertheless, this result might be the signature of an over-representation of right-handers with a right DE in a random population of right-handers, as pointed out above. This assumption is consolidated by the large inter-individual variability in splenial connectivity reported by Putnam et al. (2010). Further studies, using for instance diffusion tensor imaging as in Putnam et al. (2010), are necessary to test the hypothesis that the number of fibers that cross from the left to right hemisphere in right-handers with a left DE outnumbers the quantity of fibers that cross in the reverse direction.

Each eye projects to both hemispheres. How can we then reconcile the present results showing that IHTT is always quicker from the hemisphere ipsilateral to the DE to the other one with these hard-wired retino-cortical connections? We believe that this question could find an answer by considering the recent observations made by Shima et al. (2010). These authors found that the stimulation of the temporal hemiretina of the DE led to a greater response of the visual areas compared to the stimulation of the temporal hemiretina of the non-DE. No difference was observed between the DE and non-DE when the nasal hemiretina was stimulated. Hence these results argue for a specific link between the DE and its ipsilateral hemisphere. Accordingly, the interhemispheric transfer would be faster from the hemisphere with the larger visual activation (i.e., the ipsilateral hemisphere with respect to the DE).

### Functional considerations

The function of the visual activity propagation at the level of the posterior parietal cortices from the directly activated hemisphere to the other one remains debated (for reviews, see Schulte and Müller-Oehring, 2010; Schmidt, 2013; Bocci et al., 2014). One classically considered possibility is that this information transfer would allow the binding of the two visual hemifields, each one represented in a different hemisphere. From a more general point of view, it is also proposed that callosal projections play an active role in gain control by scaling cortical responses according to characteristics of visual afferent drive (e.g., intensity, frequency). Indeed, depending on these characteristics, either inhibitory or excitatory influences could be observed.

The question of the origin or of the functional aspect of an asymmetry in the IHTT of visual information is even more speculative. Based on the idea that the transfer of information was always faster from the right to the left hemisphere (see above), Marzi (2010) proposed that this asymmetry could be linked to the specialization of the right hemisphere for visuo-spatial attention. According to this hypothesis, this asymmetrical IHTT could be beneficial to rapidly access the left hemisphere during the myriad of cognitive and motor behaviors that are based on visuo-spatial processes. Showing that IHTT is shorter from left to right hemisphere in right-hander participants with a left DE, our findings challenge such functionality of asymmetrical communication. Further studies are required to investigate the possible functions of eye dominance.

Finally, previous works evidenced that the DE may change as a function of horizontal gaze orientation (Khan and Crawford, 2001; Carey and Hutchinson, 2013). These studies indeed show that eye dominance is strengthened when participants' gaze is oriented toward the ipsilateral visual hemifield with respect to their DE (determined with the classical methods imposing a centrally fixed gaze) but that the other eye becomes dominant when their gaze is directed toward the contralateral hemifield. The present study was not designed to evaluate this dynamical aspect of eye dominance and great care was taken to be sure that participants' gaze remained centrally fixed. Nevertheless, as a follow-up study, it would be interesting to determine if the IHTT asymmetries that we observed here is modified when the gaze is oriented toward the contralateral hemifield with respect of the DE.

## Conclusion

The present study provides the first electrophysiological evidence of a strong impact of eye dominance on the inter-hemispheric transfer of visual information in right-handers and, to a lesser extent, in left-handers. As such, it reveals important aspects of the human brain lateralization that have been largely overlooked. Although the neurophysiological basis of this eye dominance, its functional role and its impact on cognitive processes remain largely unknown and call for further investigations, the present results indicate that eye dominance needs to be considered in models of visuo-cognitive and of visuo-motor processes.

## Author contributions

Study conception and design: RC, AG; Acquisition of data: RC; Analysis and interpretation of data: RC, AG; Drafting of manuscript: RC, JB, AG.

### Conflict of interest statement

The authors declare that the research was conducted in the absence of any commercial or financial relationships that could be construed as a potential conflict of interest.

## Supplementary material

Supplementary Figure 1 Estimated source maps on the cortical surface (LORETA) based on the grand average of the 11 right-handers with a left DE after **(A)** right visual field (RVF) and **(B)** left visual field (LVF) stimulations. For each part, the left panel corresponds to the source localization at the latency of N160 peak recorded over the hemisphere contralateral to the stimulation (i.e., before interhemispheric transfer) whereas right panel shows the source localization at latency of N160 peak recorded over the contralateral hemisphere with respect to the simulation (i.e., after interhemispheric transfer). Source localization shows an interhemispheric transfer ~160–170 ms, after the visual stimulation, occurring in the medium/superior occipital gyrus (Brodmann area 19; blue areas). For the sake of clarity, only activity sources that were 12% above minimal activation are shown.

Supplementary Figure 2 Estimated source maps on the cortical surface (LORETA) based on the grand average of the 7 left-handers with a right DE after **(A)** right visual field (RVF) or **(B)** left visual field (LVF) stimulations. For each part, the left panel corresponds to the source localization at the latency of N160 peak recorded over the hemisphere contralateral to the stimulation (i.e., before interhemispheric transfer) whereas right panel shows the source localization at latency of N160 peak recorded over the contralateral hemisphere with respect to the simulation (i.e., after interhemispheric transfer). Source localization shows an interhemispheric transfer ~160–170 ms, after the visual stimulation, occurring in the medium/superior occipital gyrus (Brodmann area 19; blue areas). For the sake of clarity, only activity sources that were 14% above minimal activation are shown.

Supplementary Figure 3 Estimated source maps on the cortical surface (LORETA) based on the grand average of the 7 left-handers with a left DE following a **(A)** right visual field (RVF) or **(B)** left visual field (LVF) stimulation. For each part, the left panel corresponds to the source localization at the latency of N160 peak recorded over the hemisphere contralateral to the stimulation whereas right panel shows the source localization at latency of N160 peak recorded over the contralateral hemisphere with respect to the simulation. Source localization shows an interhemispheric transfer ~160–170 ms, after the visual stimulation, occurring in the medium/superior occipital gyrus (Brodmann area 19; blue areas). For the sake of clarity, only activity sources that were 14% above minimal activation are shown.
