# Supplementary figures and images for: Interhemispheric Transfer Time Asymmetry of Visual Information Depends on Eye Dominance: An Electrophysiological Study
Source: Front Neurosci. 2018 Feb 16;12:72. doi: 10.3389/fnins.2018.00072 (PMC5826321; doi:10.3389/fnins.2018.00072)

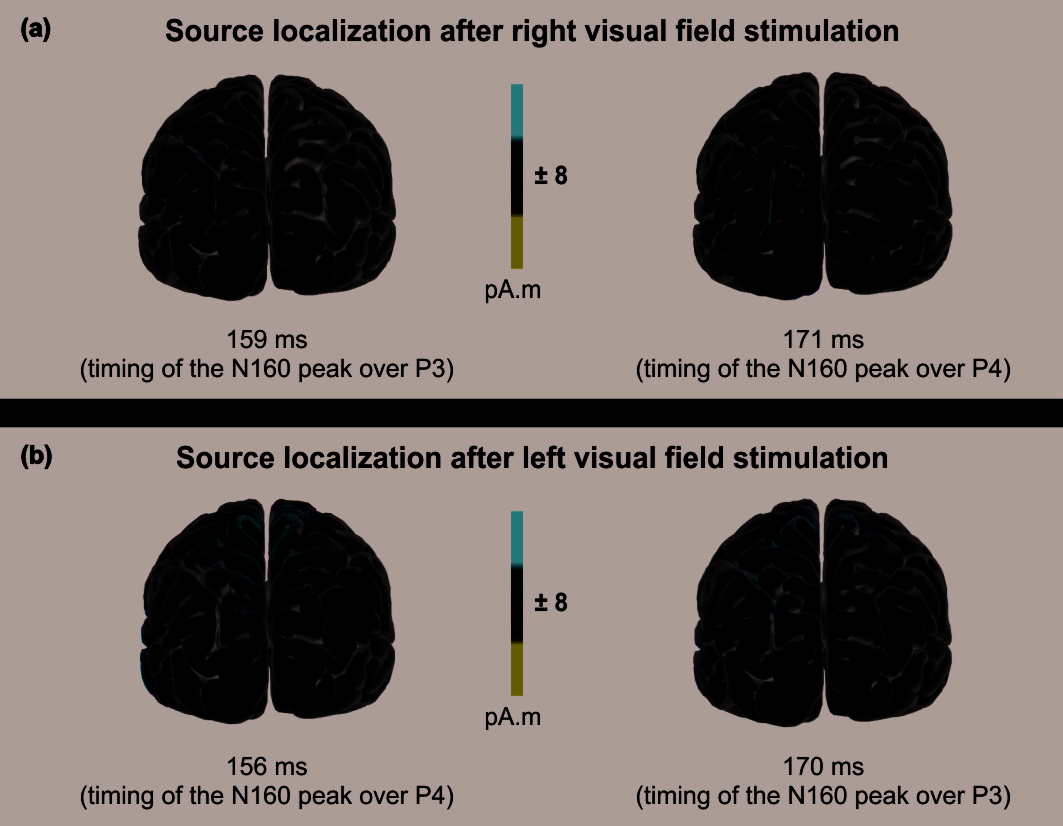

Supplement: Supplementary Figure 1 — Estimated source maps on the cortical surface (LORETA) based on the grand average of the 11 right-handers with a left DE after (A) right visual field (RVF) and (B) left visual field (LVF) stimulations. For each part, the left panel corresponds to the source localization at the latency of N160 peak recorded over the hemisphere contralateral to the stimulation (i.e., before interhemispheric transfer) whereas right panel shows the source localization at latency of N160 peak recorded over the contralateral hemisphere with respect to the simulation (i.e., after interhemispheric transfer). Source localization shows an interhemispheric transfer ~160–170 ms, after the visual stimulation, occurring in the medium/superior occipital gyrus (Brodmann area 19; blue areas). For the sake of clarity, only activity sources that were 12% above minimal activation are shown. [file Image1.TIF]

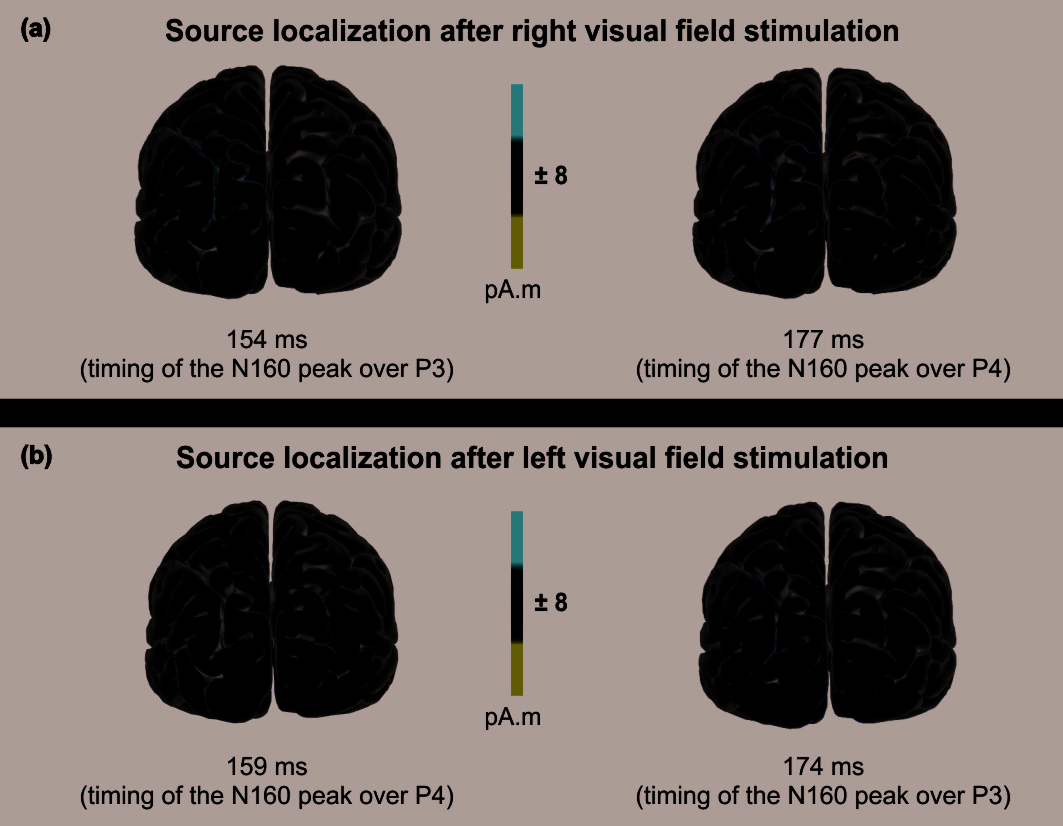

Supplement: Supplementary Figure 2 — Estimated source maps on the cortical surface (LORETA) based on the grand average of the 7 left-handers with a right DE after (A) right visual field (RVF) or (B) left visual field (LVF) stimulations. For each part, the left panel corresponds to the source localization at the latency of N160 peak recorded over the hemisphere contralateral to the stimulation (i.e., before interhemispheric transfer) whereas right panel shows the source localization at latency of N160 peak recorded over the contralateral hemisphere with respect to the simulation (i.e., after interhemispheric transfer). Source localization shows an interhemispheric transfer ~160–170 ms, after the visual stimulation, occurring in the medium/superior occipital gyrus (Brodmann area 19; blue areas). For the sake of clarity, only activity sources that were 14% above minimal activation are shown. [file Image2.TIF]

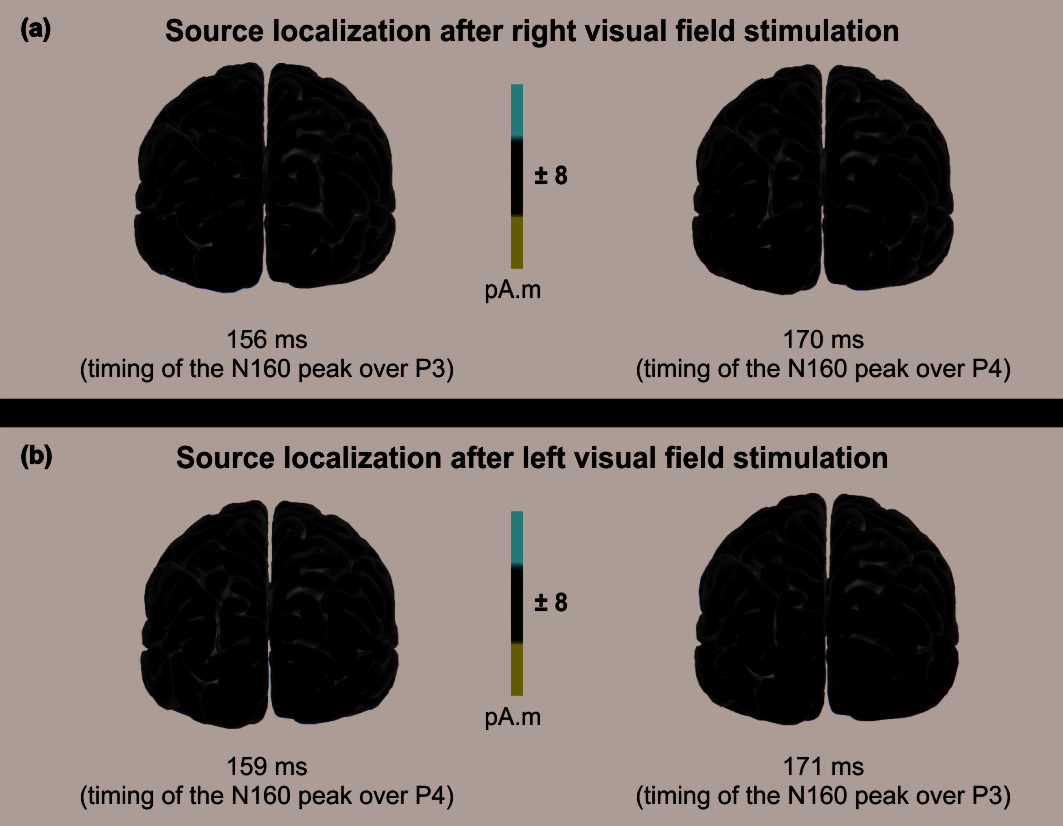

Supplement: Supplementary Figure 3 — Estimated source maps on the cortical surface (LORETA) based on the grand average of the 7 left-handers with a left DE following a (A) right visual field (RVF) or (B) left visual field (LVF) stimulation. For each part, the left panel corresponds to the source localization at the latency of N160 peak recorded over the hemisphere contralateral to the stimulation whereas right panel shows the source localization at latency of N160 peak recorded over the contralateral hemisphere with respect to the simulation. Source localization shows an interhemispheric transfer ~160–170 ms, after the visual stimulation, occurring in the medium/superior occipital gyrus (Brodmann area 19; blue areas). For the sake of clarity, only activity sources that were 14% above minimal activation are shown. [file Image3.TIF]
